# A third of the radiotracer dose: two decades of progress in pediatric [^18^F]fluorodeoxyglucose PET/CT and PET/MR imaging

**DOI:** 10.1007/s00330-023-10319-6

**Published:** 2023-10-19

**Authors:** Stephan Waelti, Stephan Skawran, Thomas Sartoretti, Moritz Schwyzer, Antonio G. Gennari, Cäcilia Mader, Valerie Treyer, Christian J. Kellenberger, Irene A. Burger, Thomas Hany, Alexander Maurer, Martin W. Huellner, Michael Messerli

**Affiliations:** 1 Department of Nuclear Medicine, University Hospital Zurich, Rämistrasse 100, CH-8091 Zurich, Switzerland; 2 University of Zurich, Zurich, Switzerland; 3 Department of Radiology and Nuclear Medicine, Children’s Hospital of Eastern Switzerland, St. Gallen, Switzerland; 4 Department of Diagnostic Imaging, University Children’s Hospital Zurich, Zurich, Switzerland; 5 Department of Nuclear Medicine, Kantonsspital Baden, Baden, Switzerland; 6 MRI Bahnhofplatz, Zurich, Switzerland

**Keywords:** [^18^F]fluorodeoxyglucose, Positron emission tomography, Pediatrics, Radioactive tracers, Radiation dose

## Abstract

**Objectives:**

To assess the evolution of administered radiotracer activity for F-18-fluorodeoxyglucose (18F-FDG) PET/CT or PET/MR in pediatric patients (0–16 years) between years 2000 and 2021.

**Methods:**

Pediatric patients (≤ 16 years) referred for 18F-FDG PET/CT or PET/MR imaging of the body during 2000 and 2021 were retrospectively included. The amount of administered radiotracer activity in megabecquerel (MBq) was recorded, and signal-to-noise ratio (SNR) was measured in the right liver lobe with a 4 cm^3^ volume of interest as an indicator for objective image quality. Descriptive statistics were computed.

**Results:**

Two hundred forty-three children and adolescents underwent a total of 466 examinations. The median injected 18F-FDG activity in MBq decreased significantly from 296 MBq in 2000–2005 to 100 MBq in 2016–2021 (*p* < 0.001), equaling approximately one-third of the initial amount. The median SNR ratio was stable during all years with 11.7 (interquartile range [IQR] 10.7–12.9, *p* = 0.133).

**Conclusions:**

Children have benefited from a massive reduction in the administered 18F-FDG dose over the past 20 years without compromising objective image quality.

**Clinical relevance statement:**

Radiotracer dose was reduced considerably over the past two decades of pediatric F-18-fluorodeoxyglucose PET/CT and PET/MR imaging highlighting the success of technical innovations in pediatric PET imaging.

**Key Points:**

*• The evolution of administered radiotracer activity for F-18-fluorodeoxyglucose (18F-FDG) PET/CT or PET/MR in pediatric patients (0–16 years) between 2000 and 2021 was assessed.*

*• The injected tracer activity decreased by 66% during the study period from 296 megabecquerel (MBq) to 100 MBq (p < 0.001).*

*• The continuous implementation of technical innovations in pediatric hybrid 18F-FDG PET has led to a steady decrease in the amount of applied radiotracer, which is particularly beneficial for children who are more sensitive to radiation.*

**Supplementary Information:**

The online version contains supplementary material available at 10.1007/s00330-023-10319-6.

## Introduction

[^18^F]Fluorodeoxyglucose ([^18^F]FDG) positron emission tomography (PET) combined with computed tomography (CT) or magnetic resonance (MR) imaging plays an important role in the diagnosis, staging, response assessment, and management of metabolically active malignancies in children, including lymphoma, malignant soft tissue and bone sarcoma, melanoma, Langerhans cell histiocytosis, and MIBG-negative neuroblastoma, as well as non-malignant diseases such as chronic granulomatous disease or fever of unknown origin [1–18]. For hybrid PET imaging, [^18^F]FDG is considered the most common radiopharmaceutical in clinical routine [19]. While hybrid [^18^F]FDG PET imaging has achieved widespread clinical adoption due to its merits in the diagnostic workup of children, its use is associated with radiation exposure. Especially for children, radiation exposure is of great concern: Firstly, recent studies suggest that children exhibit an increased radiation sensitivity for at least 25% of cancer sites, and secondly, the longer life expectancy of children increases the likelihood for secondary, radiation-induced cancers to emerge. Thus, a variety of epidemiological studies have linked childhood radiation exposure including that associated with medical imaging to the occurrence of certain cancers such as central nervous system (CNS) tumors or leukemia [20–24].

In hybrid PET, radiation exposure originates from the radiopharmaceutical [^18^F]FDG, and in the case of PET/CT also from the CT scanner [25, 26]. With PET/MR, all of the radiation exposure derives from the injected [^18^F]FDG [27–29]. Since the introduction of integrated PET/CT and later PET/MR, different technological developments in PET, such as detector and reconstruction algorithms as well as dose administration protocols, have facilitated reductions of the amount of administered radiotracer activity [4, 19].

In an effort to quantify the developments and improvements of the last two decades, we aimed to systematically analyze the evolution of administered radiotracer activity in pediatric patients who underwent [^18^F]FDG PET/CT or PET/MR in the period from 2000 to 2021 at a large tertiary medical center. Therein, we hypothesized that the technical improvements of hybrid PET imaging of the last two decades have facilitated considerable reductions in the amount of administered radiotracer activity without compromising image quality.

## Materials and methods

### Study design and population

In this retrospective single-center study, we included all pediatric patients aged 0–16 years who underwent a clinically indicated [^18^F]FDG PET/CT or PET/MR of the body between January 2000 and May 2021 at a large tertiary medical center. Clinical information including age, gender, body height and weight, International Classification of Diseases code (ICD-10), scanned body region, and amount of administered radiotracer activity in megabecquerel (MBq) were recorded. The study was approved by the local ethics committee (trial number BASEC 2020-03067) and was conducted in compliance with ICH-GCP-rules and the Declaration of Helsinki. The use for written informed consent was waived for patients scanned before January 2016. After 2016, only patients whose informed consent for further use of their medical data was available (due to a change in national regulatory rules) were included.

### PET acquisition and image reconstruction

PET/CT and PET/MRI examinations were performed on 6 different scanners of the manufacturer General Electric (GE) Healthcare, all of which were up-to-date at the time of the scan. The following scanners were used in the course of time: Discovery LS, Discovery STE, Discovery RX, Discovery 690, Discovery MI, and Signa PET/MR. A detailed overview of the scanners’ characteristics is provided in the Supplementary Material. Exams were performed using a standardized clinical [^18^F]FDG dosage protocol, until 2016 with body weight–dependent amount of radiotracer activity, from 2017 with a body weight–dependent BMI-adapted protocol in accordance with the European Association of Nuclear Medicine (EANM) consensus guidelines and the 2016 update of the North American guidelines [30, 31]. Participants fasted for at least 4 h prior to [^18^F]FDG injection. Uptake time was set to 60 min by default.

CT or MR scans for attenuation correction and anatomical localization were performed as either whole-body examination (vertex to feet, e.g., in melanoma) or partial-body examination (vertex to thighs). Following the CT or MR acquisition, PET images were acquired, covering the same anatomical region. Since the first PET scanners have been introduced, several different PET reconstruction algorithms have been used [32]. In the early days, studies were reconstructed with simple and robust conventional filtered back-projection algorithms and later with iterative reconstruction algorithms [32–34]. In this context, it should be mentioned that the implementation of more modern reconstruction algorithms can also be associated with a decrease in the amount of administered radiotracer activity. More precisely, more modern algorithms can produce an image with acceptable image noise despite a lower applied amount of radiotracer activity [35].

### Image quality

To assess objective image quality, one reader (A.G.G., 6 years of experience in radiology, and 1 year of experience in PET reading) carried out a signal-to-noise ratio (SNR) measurement in the right liver lobe in each examination, using a commercially available image analysis software (Advantage Workstation Version 4.7, GE Healthcare). Importantly, only liver parenchyma with normal appearance on both PET and CT was used for measurements. A volume of interest (VOI) with a standardized size of 4 cm^3^ was manually placed avoiding focal, non-physiologic uptake. SNR was defined as liver’s maximum standardized uptake value divided by the liver’s SUV standard deviation.

### Statistical analysis

All statistical analyses were performed using the open-source statistics software R (version 4.1.0, R Foundation for Statistical Computing) [36]. Normality of the variables was assessed using the Shapiro-Wilk test. Categorical variables are expressed as frequency distribution and were compared using Fisher’s exact test. Continuous variables are given as median with interquartile range (IQR) in parentheses. Assessment of group differences was determined using the Kruskal-Wallis test for multiple groups and Wilcoxon test for two groups. For analysis of change in administered [^18^F]FDG activity over time, linear models were fitted, and relative changes were calculated between the beginning and end of the study period. For all comparisons, a *p*-value of < 0.05 was considered to be statistically significant.

## Results

### Study population characteristics

A detailed overview of the study population is provided in Table 1. In brief, 243 children and adolescents undergoing a total of 466 examinations, including 375 PET/CT scans (81%) and 91 PET/MR scans (19%), were included. The patients underwent [^18^F]FDG PET for hematologic neoplasms (mainly lymphoma) in 45%, osseous neoplasms (mainly osteosarcoma and Ewing sarcoma) in 11%, non-neoplastic hematologic diseases (mainly chronic granulomatous disease) in 8%, fever of unknown origin in 5%, soft tissue neoplasms (mainly rhabdomyosarcoma) in 5%, melanoma in 4%, osteomyelitis (infectious and non-infectious) in 3%, urogenital neoplasms in 3%, gastrointestinal neoplasms in 2%, and other diseases in 14% of cases, respectively. Sixty-three examinations were performed on a Discovery LS, 88 on a Discovery STE, 161 on a Discovery RX, 33 on a Discovery 690 VCT, 30 on a Discovery MI Gen 2 with digital detectors, and 91 on a Signa PET/MR.

**Table 1:** Demographic data of study subjects (*n* = 243) undergoing [^18^F]FDG PET/CT (*n* = 375) or PET/MR (*n* = 91)

Female/male, *n* (%)	104 (43%)/139 (57%)
Age at scan, years	14 (11–15) with an age range of 0–16
Body weight at scan, kg	45 (30–59)
Body height at scan, m	1.59 (0.68–1.89)
BMI, kg/m^2^	17.6 (15.0–20.8)
Injected FDG activity, MBq	218 (132–301)
Injected FDG activity per body weight, MBq/kg	5.3 (3.0–7.0)
SNR (liver)	11.7 (10.7–12.9)

Values are given as absolute numbers and percentages in parenthesis or as median (25th to 75th percentile)

*BMI*, body mass index; *kg*, kilogram; *MBq*, megabecquerel; *PET/CT*, positron emission tomography/computed tomography

### Evolution of administered radiotracer activity

The median injected [^18^F]FDG activity was 218 MBq (range 32–402 MBq). From the time period 2000 to 2005 to the time period 2016–2021, the injected tracer activity decreased significantly (*p* < 0.001) by 66% from 296 MBq (IQR 253–372 MBq) to 100 MBq (IQR 79–161 MBq). Patient’s age-based, year-based, and scanner-based analysis of the study population are given in Tables 2, 3, and 4.

**Table 2:** Age group–based analysis of administered [^18^F]FDG activity

Age group–based analysis					
	0–5 years *n* = 29	6–8 years *n* = 45	9–12 years *n* = 109	13–16 years *n* = 283	*p*-value
Body weight, kg	15 (12–17)	22 (19–26)	32 (27–41)	56 (45–65)	< 0.001
Body height, m	0.99 (0.91–1.03)	1.21 (1.14–1.28)	1.45 (1.37–1.52)	1.69 (1.60–1.76)	< 0.001
BMI, kg/m^2^	15.4 (14.2–16.8)	14.7 (13.8–16.8)	16.0 (14.3–18.1)	19.1 (16.13–21.86)	< 0.001
Administered activity, MBq	100 (71–128)	142 (89–177)	162 (81–223)	280 (198–322)	< 0.001
Administered activity per body weight, MBq/kg	7.7 (4.4–9.1)	6.9 (6.0–7.6)	3.9 (2.7–7.3)	5.1 (3.1–6.4)	< 0.001
Administered activity reduction^a^	− 57%	− 80%	− 84%	− 79%	-
SNR (liver)	12.3 (10.7–13.2)	11.6 (10.6–12.7)	11.8 (11.0–13.3)	11.7 (10.6–12.7)	0.265

Values are given as absolute numbers and percentages in parenthesis or as median (25th to 75th percentile)

*BMI*, body mass index; *kg*, kilogram; *MBq*, megabecquerel; *SNR*, signal-to-noise ratio

^a^Calculated as relative % difference of the estimated values at the beginning (January 2000) and end of the study period (May 2021), using a linear model

**Table 3:** Year-based analysis of administered [^18^F]FDG activity

Year-based analysis					
	2000–2005 *n* = 32	2006–2010 *n* = 165	2011–2015 *n* = 124	2016–2021 *n* = 145	*p*-value
Body weight, kg	37 (30–47)	42 (29–56)	46 (33–61)	52 (30–65)	0.009
Body height, m	1.55 (1.44–1.65)	1.62 (1.39–1.73)	1.58 (1.44–1.71)	1.58 (1.40–1.71)	0.579
BMI, kg/m^2^	15.9 (14.6–18.2)	16.0 (14.3–18.9)	18.5 (15.7–22.2)	18.78 (16.1–22.1)	< 0.001
Administered activity, MBq	296 (253–327)	297 (208–326)	223 (178–290)	100 (79–161)	< 0.001
Administered activity per body weight, MBq/kg	7.3 (5.6–8.9)	6.7 (5.7–7.9)	5.2 (3.9–6.5)	2.7 (2.0–3.1)	< 0.001
Administered activity reduction^a^	*Reference*	− 42%	− 60%	− 83%	-
SNR (liver)	11.5 (10.4–12.2)	11.9 (10.9–13.3)	11.6 (10.7–12.7)	11.5 (10.7–12.7)	0.133

Values are given as absolute numbers and percentages in parenthesis or as median (25th to 75th percentile)

*BMI*, body mass index; *kg*, kilogram; *MBq*, megabecquerel; *SNR*, signal-to-noise ratio

**Table 4:** Scanner-based analysis of administered [^18^F]FDG activity

Scanner-based analysis							
	Discovery LS *n* = 63	Discovery STE *n* = 88	Discovery RX *n* = 161	Discovery 690 VCT *n* = 33	Discovery MI *n* = 30	Signa PET/MR *n* = 91	*p*-value
Body weight, kg	38 (29–50)	42 (27–58)	42 (30–57)	60 (53–69)	43 (25–52)	52 (32–65)	< 0.001
Body height, m	1.57 (1.41–1.70)	1.55 (1.32–1.72)	1.58 (1.37–1.72)	1.68 (1.60–1.71)	1.58 (1.27–1.68)	1.57 (1.45–1.74)	0.101
BMI, kg/m^2^	15.6 (14.2–17.7)	20.3 (16.2–22.9)	17.1 (14.9–19.6)	21.8 (18.8–24.0)	17.4 (15.7–18.9)	20.3 (16.2–22.9)	< 0.001
Administered activity, MBq	300 (249–326)	260 (195–312)	266 (186–323)	191 (161–231)	83 (71–97)	103 (79–153)	< 0.001
Administered activity per body weight, MBq/kg	7.5 (5.7–8.8)	6.1 (5.1–7.5)	6.2 (5.0–7.2)	3.0 (3.0–3.2)	2.0 (1.9–2.9)	2.4 (2.0–3.0)	< 0.001
Administered activity reduction^a^	-	− 19%	− 17%	− 60%	−73%	−68%	-
SNR (liver)	11.5 (10.6–12.2)	11.8 (10.6–12.9)	12.0 (11.1–13.3)	10.7 (10.3–11.9)	12.7 (11.6–13.7)	11.3 (10.8–12.4)	< 0.001

Values are given as absolute numbers and percentages in parenthesis or as median (25th to 75th percentile)

*BMI*, body mass index; *kg*, kilogram; *MBq*, megabecquerel; *SNR*, signal-to-noise ratio

^a^Calculated as relative % difference of the estimated values compared to the scanner used at the beginning of the study period (Discovery LS)

**Fig. 1:**
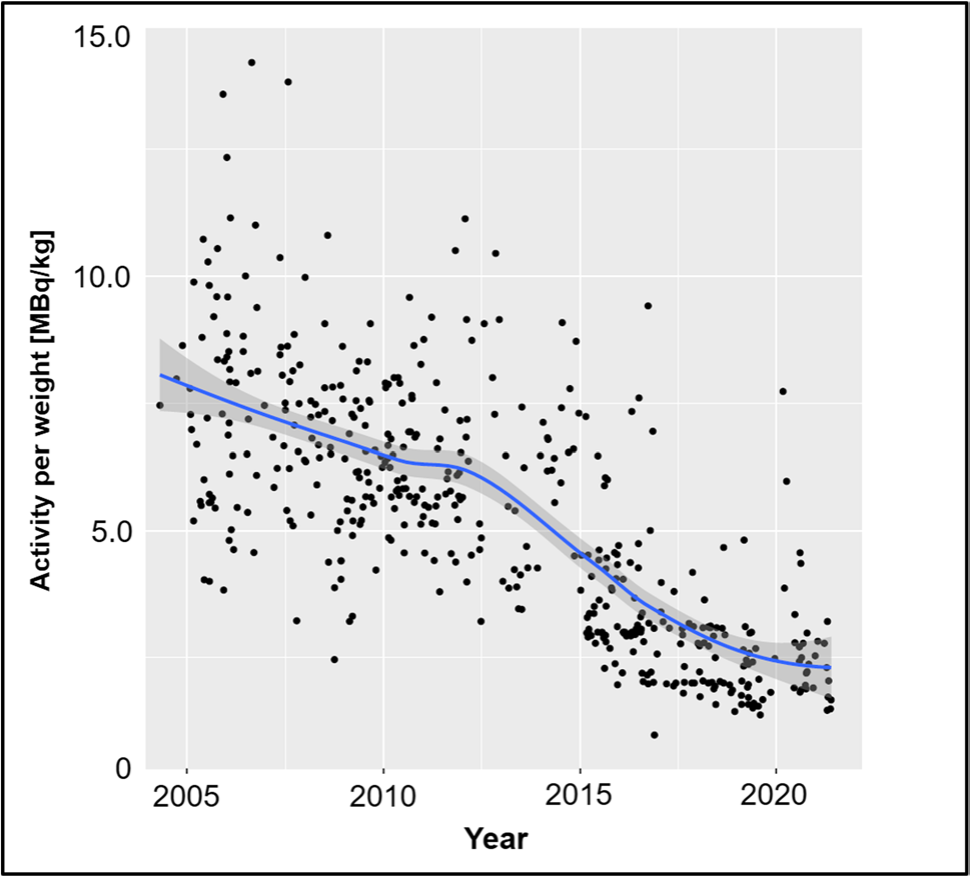
Overview of the amount of injected activity between 2000 and 2021 with a linear regression line and the corresponding *R*^2^ value for a linearly fitted model. Each individual point represents one scan

**Fig. 2:**
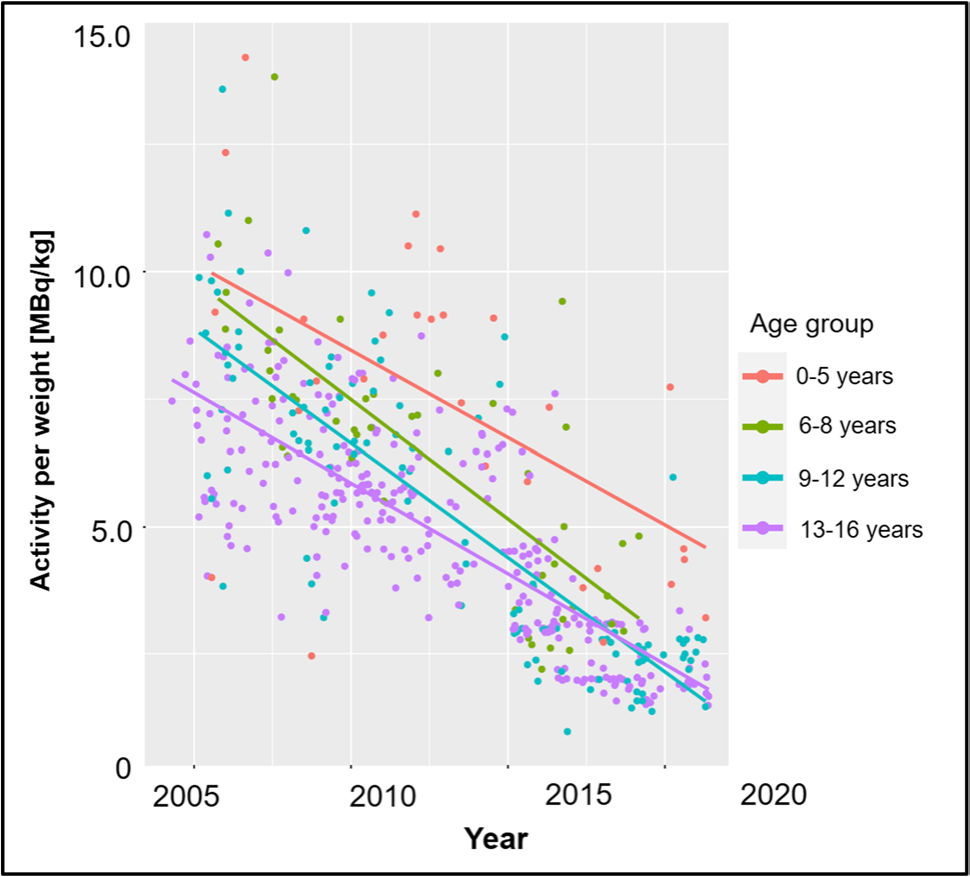
Overview of the amount of injected activity between 2000 and 2021, divided into four age groups. Each individual point represents one scan

**Fig. 3:**
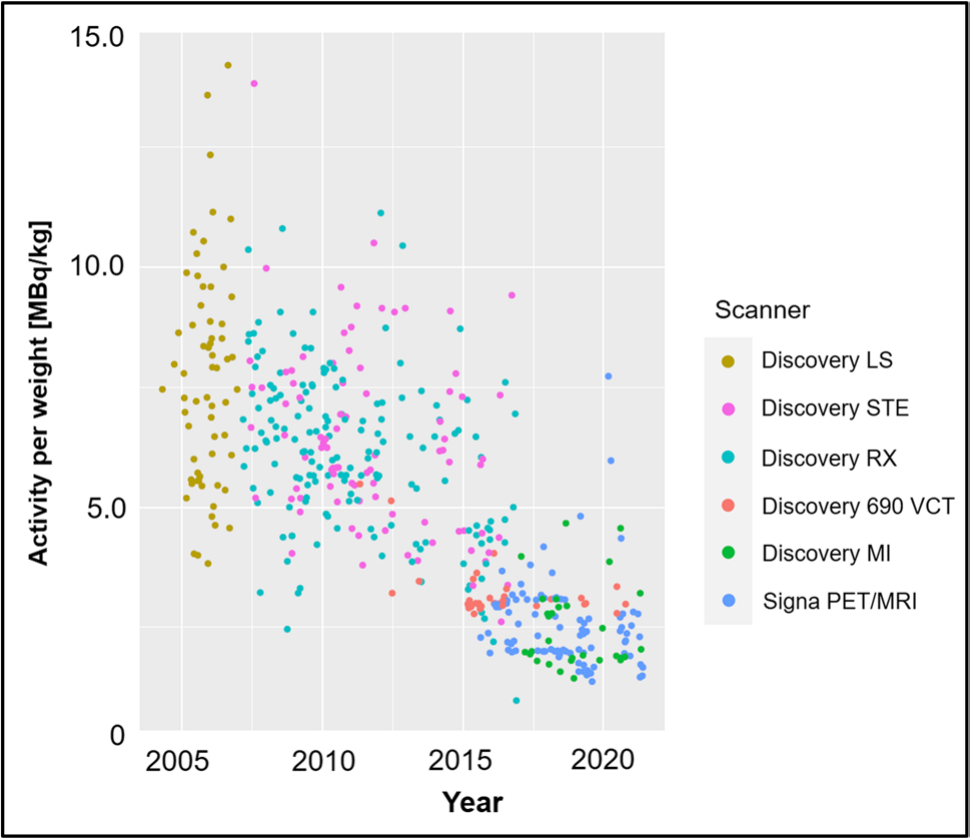
Overview of the amount of injected activity between 2000 and 2021, divided into six groups depending on the scanner used. Each individual point represents one scan

The overall evolution of the administered [^18^F]FDG radiotracer activity over time is given in Fig. 1. An age group–based analysis is presented in Fig. 2. The amount of [^18^F]FDG decreased continuously and substantially over time and for all age groups. Figure 3 illustrates the amount of injected activity between 2000 and 2021 divided into groups depending on the scanner used.

### Signal-to-noise ratio of PET

**Fig. 4:**
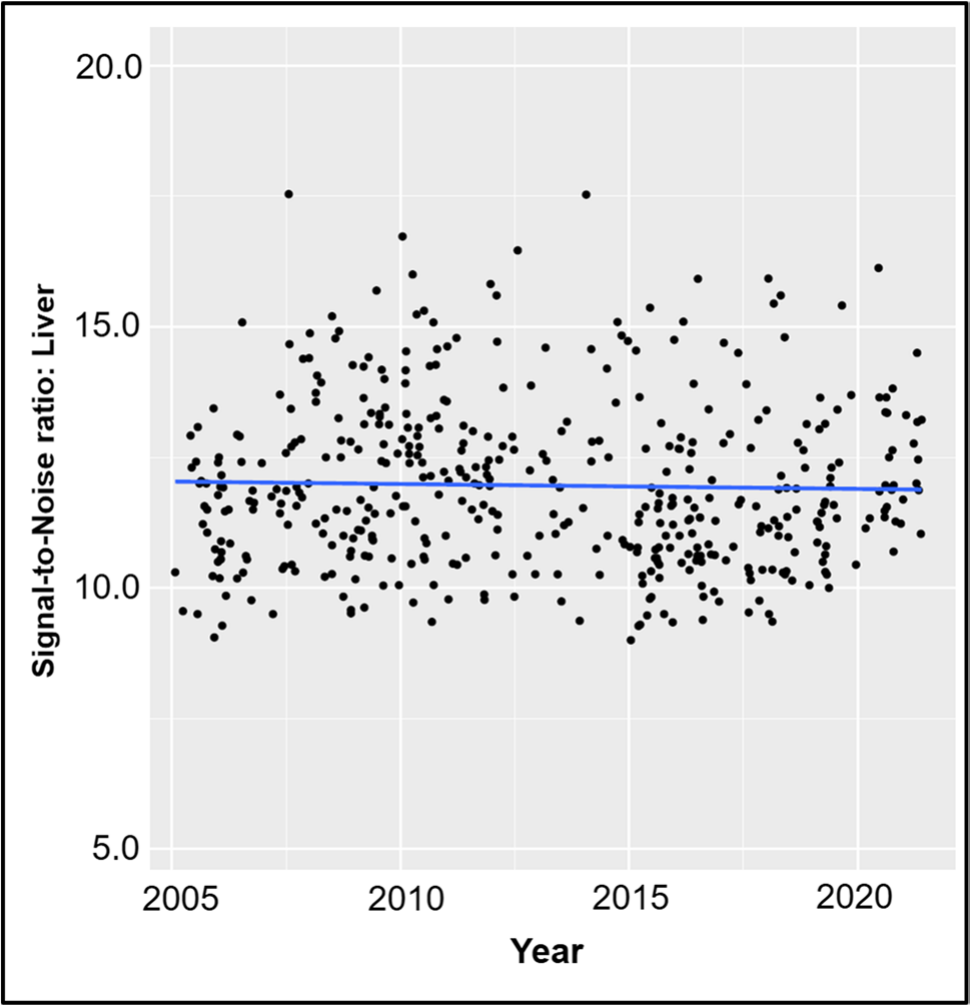
Signal-to-noise (SNR) measured in the liver for each scan (*n* = 466). The blue line represents the median SNR

The median SNR was 11.7 (IQR 10.7–12.9) and was stable during all years, as shown in Fig. 4. Thus, the decreasing amount of injected activity did not result in a lower SNR.

## Discussion

In this study, we assessed the evolution of the amount of injected radiotracer activity of [^18^F]FDG in pediatric hybrid PET. The major findings of our study can be summarized as follows: First, we observed a major reduction in the amount of administered [^18^F]FDG activity in the last 20 years. Second, the reduction of [^18^F]FDG activity over time ranged from −57% up to −84%, depending on the age group, with the smallest reduction observed in the youngest patients. Third, the median administered [^18^F]FDG activity dropped from 7.3 MBq/kg in 2000–2005 to 2.7 MBq/kg in 2016–2021, respectively. Fourth, signal-to-noise ratio, an objective surrogate for image quality, remained stable during the same time.

Accurate staging and re-staging of oncological diseases in children and adolescents are crucial for appropriate treatment management and optimal outcomes. [^18^F]FDG PET/CT and PET/MR have evolved as important tools in the diagnosis, staging, and response assessment of various oncological diseases. Unfortunately, the use of PET/CT—and to a lesser extent PET/MR—is associated with considerable radiation exposure, which is particularly concerning for children given their increased sensitivity towards [37].

Concerning pediatric hybrid PET imaging, the EANM published a pediatric dosage card with respect to the amount of FDG in 2007 [38]. This guideline included recommendations for both “baseline activity” (for calculation purposes) and “minimum recommended activity” with multiplication factors. Between 2008 and 2016, these guidelines were updated repeatedly with the EANM further reducing the recommended value for the “minimum recommended activity” [31, 39]. These recommendations were also used at our institution, but with each hardware and software innovation implemented on our imaging systems the injected dose was reduced even further to fully harness the capabilities of these technological innovations.

These efforts were made in accordance with the ALARA principle (i.e., as low as reasonably achievable) to protect this radiation-sensitive patient cohort as much as possible. Nonetheless, care was given not to compromise image quality or the diagnostic evaluability of the acquired scans.

Specifically, the introduction of weight-adjusted tracer activity administration at our institution in 2016 marked an important milestone with it resulting in a significant reduction in patient’s exposure. Importantly, this dose regimens achieved widespread adoption at our institution beyond hybrid PET imaging. Notably, for cardiac SPECT imaging in adults, the weight-adjusted activity administration of radiotracer in combination with iterative reconstruction algorithms enabled dose reductions of 30% or even 40% [40].

Our data provides evidence that the continuous implementation of the latest hardware and software and the continuous optimization of injection protocols have enabled considerable dose reductions over the past two decades. This trend of ever decreasing radiopharmaceutical doses will continue in the next few years with exciting technologies such as artificial intelligence–powered image reconstructions entering the clinical market. Beyond these developments, scientists strive towards further improving pediatric dosage regimens. Notably, Cox et al recently investigated the relationship between patient-dependent parameters and [^18^F]FDG PET image quality in an effort to develop dedicated pediatric dosage protocols [41]. In their study of 102 children, they found that body weight was strongly associated with [^18^F]FDG PET image quality in children. Therefore, they proposed a nonlinear dosage regimen based on body mass, in order to provide a constant and clinically sufficient image quality while significantly reducing the effective dose beyond that recommended in current guidelines.

Importantly, with regard to hard- and software innovations for PET imaging, considerable advances have been made within the past 20 years. Optimizations in the scintillator crystal and photodetector combination, the acquisition electronics and advances in data processing, reconstruction, and image analysis together with increasing axial coverage of the scanner have facilitated considerable improvements in terms of the system sensitivity. The ultimate goal is always to maximize the number of counts acquired per unit of radiation dose to the patient and to achieve high spatial and temporal resolution.

These technical improvements can also be found in the scanner models used in this study [42]. For example, the earliest scanner model (Discovery LS) used 1st-generation bismuth germanate (BGO) detectors while the more recent scanner models (Discovery MI and Signa PET/MR system) use more advanced lutetium oxyorthosilicate detectors with small amounts of yttrium (LYSO) coupled with silicon photomultipliers (SiPMs). Moreover, the axial field of view increased from 15.2 cm for the oldest scanner model to ca. 25 cm for the most recent scanner models. Together with other technical improvements, these optimizations have facilitated an increase in sensitivity (in cps/kBq) from 6.41 to over 20 from the oldest to the most recent scanner models used in the current study.

Importantly, further advancements in PET instrumentation are already on the horizon as exemplified by the emergence of total body (TB) PET/CT systems. These revolutionary systems, with an extended axial field of view of up to 194 cm, enable the simultaneous imaging of the entire body. By encompassing the patient’s full anatomy, these imaging systems significantly improve signal detection efficiency, offering benefits for pediatric PET imaging. They allow for shorter acquisition times (less than a minute), reduced administered activity (as low as one-twentieth of the standard dose), and enhanced image quality due to improved signal-to-noise ratios. Moreover, this enables whole-body dynamic imaging with a temporal resolution of less than 1 s per imaging frame. Consequently, the integration of total body PET imaging into routine practice holds great potential for improving the safety and efficacy of pediatric PET procedures across a range of disorders affecting this vulnerable population [43].

Our study has some limitations. First, this study is a single-center retrospective analysis with a limited number of patients. Second, due to the long study period of over 20 years, we were not able to collect and report some data such as each PET system’s hardware and software configuration at the time of the acquisition, reconstruction methods, duration of the scan, and details on the CT and MR acquisition. Third, all examinations were performed on imaging systems of a single vendor. This may limit the generalizability of our results. Fourth, to quantify objective image quality, we only computed liver SNR. However, we acknowledge that there are many more metrics to objectify image quality. Fifth, radiation exposure from CT, which accounts for an important amount of the total radiation exposure, was not investigated.

## Conclusion

The technical innovations of the past two decades have allowed for a continuous reduction in the amount of administered [^18^F]FDG (up to 84%) in pediatric PET/CT and PET/MR. Specifically, in pediatric patients, the average amount of administered activity in 2016–2021 is only approximately one-third of the amount administered between 2000 and 2005. In light of the increased sensitivity of children towards ionizing radiation, our findings are of special clinical significance and should promote further scientific efforts towards enabling further dose reductions for clinical pediatric hybrid PET imaging.

### Supplementary Information

Below is the link to the electronic supplementary material.

Supplementary file1 (PDF 50 KB)

## Abbreviations

18F-FDG: [^18^F]fluorodeoxyglucose
ALARA: As low as reasonably achievable
CT: Computed tomography
EANM: European Association of Nuclear Medicine
ICD: International Classification of Diseases
IQR: Interquartile range
MBq: Megabecquerel
MRI: Magnetic resonance imaging
PET: Positron emission tomography
SUV_max_: Maximum standardized uptake value
TOF: Time of flight

## Declarations

### Guarantor

The scientific guarantor of this publication is PD Dr. Michael Messerli.

### Statistics and biometry

No complex statistical methods were necessary for this paper.

### Study subjects or cohorts overlap

No study subjects or cohorts have been previously reported.

### Methodology

• Retrospective

• Observational

• Performed at one institution
