# Supplementary material for: A third of the radiotracer dose: two decades of progress in pediatric [18F]fluorodeoxyglucose PET/CT and PET/MR imaging
Source: Eur Radiol. 2023 Oct 19;34(5):3252–9. doi: 10.1007/s00330-023-10319-6 (PMC11126459; doi:10.1007/s00330-023-10319-6)
Supplement: Supplementary file 1 — Supplementary file1 (PDF 50 KB) [file 330_2023_10319_MOESM1_ESM.pdf]

A third of the radiotracer dose: Two decades of progress in pediatric 18F-Fluorodeoxyglucose PET/CT and PET/MR imaging

Electronic Supplementary Material (ESM)

Eur Radiol (2023) Waelti S, Skawran S, Sartoretti T et al

	Discovery LS	Discovery STE/VCT	Discovery RX	Discovery 690 VCT	Discovery MI Gen 2 6 Ring (5-Ring)	Signa PET/MR
Scintillator material	1st Gen BGO	2nd Gen BGO	LYSO	LYSO	SiPM LYSO	SiPM LYSO
Amplifier						
Number of rings	18	24 * 560	24 * 630 crystals		45	
Scintillator dimensions (mm3)	4x8x30	6.3x4.7x30	4.2 x 6.3 x 30	4.2 x 6.3 x 25	3.95 x 5.3 x 30 (25)	40. x 5.3 x 25
Axial FOV (cm)	15.2	15.7	15.7	15.7	30 (25)	25
Transaxial FOV (cm)		70	70	70	70	60
Overlap (%)				Min 5 slices, 10%		
Sensitivity (cps/kBq)	1.27 (2D), 6.41 (3D)	2.2 (2D), 8.4 (3D)	1.7 (2D), 7.3 (3D)	7.0 / 7.5	32.64 (22.01)	21.5
Sensitivity/mm (cps/kBq*mm)		8.8	7.3	0.045	0.084	
Effective Sensitivity (cps/kBq)					150	
Scatter fraction (%)		33.9 (3D)	13.1 (2D), 31.8 (3D)	37	40.21 (41)	44.1
Peak NECR (kcps @ kBq/mL)	125 @ 58 (2D)<br>19.2 @ 7.15 (3D)	67.6 @ 12.1 (3D)	155 @ 92.1 (2D), 117.7 @ 21.7 (3D)	130 @ 29.5	434.3 @ 23.6 (312.9 @ 22.5)	212.2 @ 18.1
Axial full width at half maximum @ 1 cm (mm)		5.18 (3D)	4.8 (2D), 5.8 (3D)	5.6 (5.0 VUE Point HD)	4.26 (4.33) (VUE Point), 3.09 (3.16) (QClear)	5.34 (FBP), 4.67 (iterative)
Transaxial full width at half maximum @ 1 cm (mm)		5.12 (3D)	5.1 (2D), 5.0 (3D)	4.9 (4.0 VUE Point HD)	3.9 (VUE Point), 2.1 (QClear)	4.10 (FBP) 3.43 (iterative)

Eur Radiol (2023) Waelti S, Skawran S, Sartoretti T et al

Coincidence window width (ns)	12.5	9.3	6.5	4.9	5.25	4.57
Timing resolution (ps)<br>@ 0kBq/ml / @5.3kBq/ml			544.3	390 or 544.3	389.6 (391.6) / 407.6 (408.5)	390
Lower energy threshold (keV)	300	4.25	425	425	425	425
Slice thickness (mm)	4.25					
Image planes in the axial FOV	35			47		

**Empty fields are either due to lack of applicability (for example lack of TOF capability in earlier scanner models) or lack of data due to different NEMA standards.
